# We remember: C. Richard Conti, MD

**DOI:** 10.1002/clc.23822

**Published:** 2022-05-06

**Authors:** Nanette K. Wenger

**Affiliations:** ^1^ Emory University School of Medicine Atlanta Georgia USA; ^2^ Emory Heart and Vascular Center Atlanta Georgia USA; ^3^ Emory Women's Heart Center Atlanta Georgia USA

Dr. C. Richard Conti, a legendary cardiovascular educator, researcher, and mentor, died suddenly on February 21, 2022, in Gainesville, FL.

Dr. Conti was born in 1934 in Bethlehem, PA, graduated from Lehigh University Phi Beta Kappa in 1956 and from the Johns Hopkins University Medical School in 1960. After service in the US Army Medical Corps, Dr. Conti returned to Johns Hopkins for residency in Internal Medicine and Fellowship in Cardiology. His interest was in ischemic heart disease, and he was among the pioneers in interventional cardiology. He chaired the National Heart Lung and Blood Institute (NHLBI) first national randomized trial of medical versus surgical intervention in patients with unstable angina and chaired the Asymptomatic Ischemia Pilot Trial comparing medical therapy with revascularization in patients with stable angina. In 1974, Dr. Conti was recruited to become Chief of Cardiology at the University of Florida in Gainesville. There, as Professor of Medicine, he guided the university, to become nationally and internationally recognized as a center of excellence in cardiovascular research and training. Dr. Conti was a lifelong teacher and learner, chairing the American College of Cardiology (ACC) Self‐Study Education Materials Committee, which served to create the ACC Self‐Assessment Program (ACCSAP). As well, he contributed as Editor in Chief of ACCEL, the audio journal of ACC. In 1989−1990, Dr. Conti served as President of the American College of Cardiology.

A passionate educator, he was involved in the early efforts of the American College of Cardiology and ESC international programs, guiding the development and growth of the annual Great Wall International Conference in Cardiology in Beijing. After serving as Chief of Cardiology for 24 years, Dr. Conti relinquished this role in 1998 but remained active directing the Ambulatory Cardiology section at the University of Florida.

As well, Dr. Conti served on the subspecialty board of Cardiovascular Disease of the American Board of Internal Medicine and the Cardiology Advisory Committee of the NHLBI. He was recognized for his skill as a teacher and scholar by the British Cardiac Society, the French Cardiac Society, the European Society of Cardiology, the College of Medicine South Africa, and the Venezuelan Cardiac Society among others. He took particular pride in his daughter Jamie, a cardiologist and electrophysiologist at the University of Florida as she progressed to lead the Division of Cardiology. He received the Docteur Honoiris Causa from the Universite de Marseilles in 2000. Dick was a devoted husband, father, grandfather, and great‐grandfather. He was a marathoner and loved to golf, to ski, and to travel, taking his family on cruises worldwide. My husband Julius and I were privileged to join Dick and his lovely wife Ruth on a number of international teaching trips, where he was always the center of vibrant discussions and masterful teaching. Dick mentored many of the current leaders in cardiovascular medicine worldwide and was a personification of the concept of lifelong learning.

Dr. Conti authored or coauthored over 600 scientific manuscripts and book chapters and was editor of Clinical Cardiology from 1988 to 2011. He took a strong and enduring interest in the journal, working with Willis Hurst and Bob Schlant and many other giants of cardiology. All of us who joined with him at the editorial board meetings that he hosted every year at the American College of Cardiology will always remember these wonderful occasions.

